# Morphology-dependent field emission properties and wetting behavior of ZnO nanowire arrays

**DOI:** 10.1186/1556-276X-6-74

**Published:** 2011-01-12

**Authors:** Lujun Yao, Maojun Zheng, Li Ma, Wei Li, Mei Li, Wenzhong Shen

**Affiliations:** 1 Laboratory of Condensed Matter Spectroscopy and Opto-Electronic Physics, Department of Physics, Shanghai Jiao Tong University, Shanghai 200240, People's Republic of China; 2 Key laboratory of Artificial Structures and Quantum Control (Ministry of Education), Department of Physics, Shanghai Jiao Tong University, Shanghai 200240, People's Republic of China; 3 School of Chemistry & Chemical Technology, Shanghai Jiao Tong University, Shanghai 200240, People's Republic of China

## Abstract

The fabrication of three kinds of ZnO nanowire arrays with different structural parameters over Au-coated silicon (100) by facile thermal evaporation of ZnS precursor is reported, and the growth mechanism are proposed based on structural analysis. Field emission (FE) properties and wetting behavior were revealed to be strongly morphology dependent. The nanowire arrays in small diameter and high aspect ratio exhibited the best FE performance showing a low turn-on field (4.1 V/μm) and a high field-enhancement factor (1745.8). The result also confirmed that keeping large air within the films was an effective way to obtain super water-repellent properties. This study indicates that the preparation of ZnO nanowire arrays in an optimum structural model is crucial to FE efficiency and wetting behavior.

## Introduction

ZnO has been considered as one of the most important electronic and photonic material because of its wide direct bandgap (3.37 eV) and large exciton binding energy (60 meV). Extensive researches have been developed on the growth of quasi one-dimensional (1D) ZnO nanostructures [1,2] including nanowires, nanotubes, nanobelts, and nanoneedles. Meanwhile, these 1D ZnO nanostructures have been widely applied as room temperature UV detector [3], transparent conductive electrodes [4], sensors [1,5-7], and solar cells [8]. Recently, various inorganic semiconductor nanostructures have been the focus of the researches on the studies of FE properties [9] and wetting behavior [10], including the well-aligned 1D ZnO nanostructured arrays which have attracted great attention as promising field emission (FE) sources [1,11-14] due to their negative electron affinity [15], chemical stability, tip geometry, or apex structure. A crucial factor to influence FE performance includes the interspacing between individual nanowires or nanorods, and aspect ratio. The manner in which these structural parameters could be controlled during self-organized growth processes has developed into a challenging and technological problem for nanostructure fabrication. Too closely and too densely spaced nanostructures are both not favorable to construct FE nanodevices. On the other hand, another significant application of ZnO related to the geometric effects is the wettability [16,17], which might bring great advantages in a wide variety of applications in daily life, industry, and agriculture. The vertically aligned nanostructures involving a large amount of trapped air within the films and their high roughness have been proved to be potential for the building of hydrophobic surfaces, various surfaces of ZnO nanostructured arrays showing lotus-like water-repellent properties have been prepared in the past years [16,18,19].

However, many previous efforts in the large-scale fabrication of ZnO nanowire or nanorod arrays have been achieved by physical evaporation of the mixture of ZnO and graphite powders, chemical vapor deposition using Zn powder as the source materials, or low-temperature hydrothermal synthesis with the pre-prepared colloidal ZnO nanocrystals as the grown seeds. In this article, a novel fabrication of ZnO nanowire arrays with different structural parameters over Au-coated silicon (100) by facile thermal evaporation of ZnS precursors is reported. The nanowire diameter and growth speed were controlled by changing the thickness of coated Au film layer together with substrate locations. The authors studied the morphology-dependent FE performance, and first revealed that wetting behavior of ZnO nanowire arrays in different void ratios, which confirmed that a large amount of air kept within the films would be an effective way to obtain super water-repellent properties.

## Experimental

The fabrication was performed using a two-end open quartz tube connected to a rotary vacuum pump and a gas inlet through a vacuum coupling. The silicon (100) substrates prepared for samples A, B, and C were sonicated in acetone, washed with de-ionized (DI) water, and dried with nitrogen. Then, Au film layers were deposited on these substrates by ion sputtering from the Au target (99.999%) using an ion sputter coater (Hitachi E-1045, Hitachi Co., Tokyo, Japan.). The target-substrate distance was about 30 mm, and the pressure of sputtering chamber was pumped down to 6 Pa before deposition. The coating rate depending on discharge current was kept at 6 nm/min. The three kinds of above-mentioned substrates were sputtered for 50, 50, and 15 s, respectively. The corresponding thicknesses of Au film layers are about 50, 50, and 15 Å. Growth procedures were conducted by thermal evaporation of commercially available high purity ZnS powder and graphite powder with equal molar ratio, which was placed at the center of the quartz tube furnace. Silicon substrates were placed downstream about 5 cm (samples B and C) and upstream about 5 cm (sample A) away from the source materials to collect the products. Subsequently, we introduced an Ar gas flow of 80 sccm, and a fixed pressure at about 150 Torr was applied. The tube furnace was then heated to 750°C quickly and maintained at this peak for 30 min. After it cooled down naturally to room temperature, all the substrates appeared dark gray indicating the deposition.

The morphology and crystal structures were characterized by field emission scanning electron microscope (FE-SEM, Philips Sirion 200) and X-ray diffractometer (Bruker-AXS system) with Cu Kα radiation (λ = 1.5406 Å). The surface chemical composition of these ZnO nanowire arrays was analyzed by XPS (Kratos AXIS Ultra DLD) with a power of 150 W. A monochromatic Al Kα X-ray source (1486.6 eV) was operated in a constant analyzer energy mode. Water contact angle (CA) and sliding angle were measured using an optical contact-angle meter system (Data Physics Instrument GmbH, Germany) at ambient temperature. FE properties were carried out employing a two-parallel-plate configuration in an ultrahigh vacuum chamber (5 × 10^-^^7 ^Pa). In brief, samples were stuck onto a stainless-steel sample stage using conducting glue to act as the cathode, while another parallel stainless steel plate served as the anode with a fixed cathode-anode distance of 300 μm. The emission current was monitored via a Keithley 485 picoammeter.

## Results and discussions

### Structural and compositional characterization of ZnO nanowire arrays

**Figure 1:**
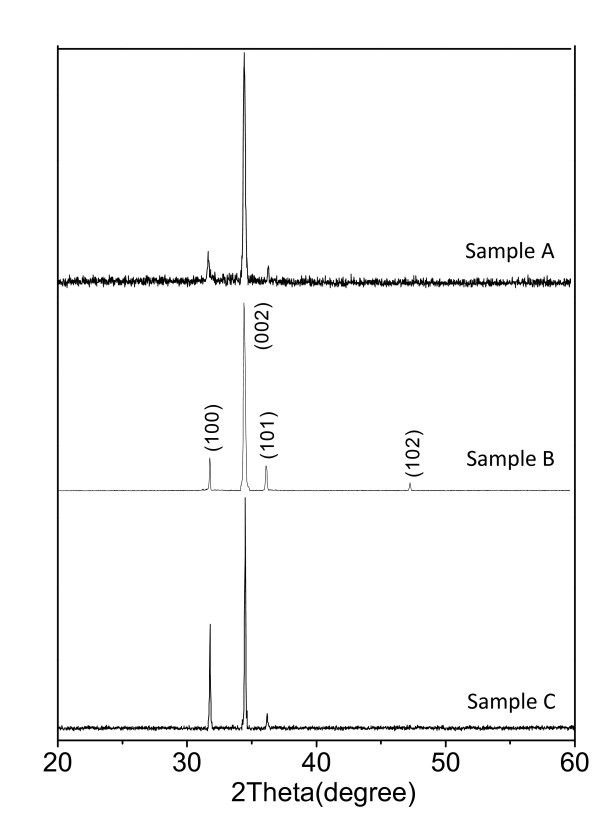
**XRD patterns of the three kinds of ZnO nanowire arrays**.

Figure 1 shows the X-ray diffraction patterns used to assess the overall structure and phase purity. All positions of the peaks can be readily indexed to the hexagonal wurtzite ZnO with lattice constants *a *= 3.25 Å and *c *= 5.21 Å (JCPDS card No. 80-0075). In particular, we can see that (002) peak located at about 34.4° is much stronger than the others for all of the three samples, which means these nanowire arrays have a preferential orientations in the *c*-axis direction. Moreover, it is clearly seen that the peak intensity of sample B is the strongest among the three products, whereas the sample A has the weakest peak intensity. The reason can be attributed to ZnO film thickness as well as their void ratios, which can be obtained from Table 1. The sample B which has a thick film with small void ratio shows higher peak intensity than the other two samples.

**Table 1:** The structural parameters of the three kinds of nanowire arrays

Sample	Diameter (nm)	Length (μm)	Aspect ratio	**Density (μm**^**-2**^**)**	Void ratio (%)	***E***_**to **_**(V/μm)**	β	CA
A	300	6	20	1.3	90.8	8.4	1209.5	142.1 ± 1°
B	600	25	41.7	0.57	83.9	5.8	1566.7	94.8 ± 1°
C	80	25	312.5	4.2	97.9	4.1	1745.8	154.3 ± 1°

**Figure 2:**
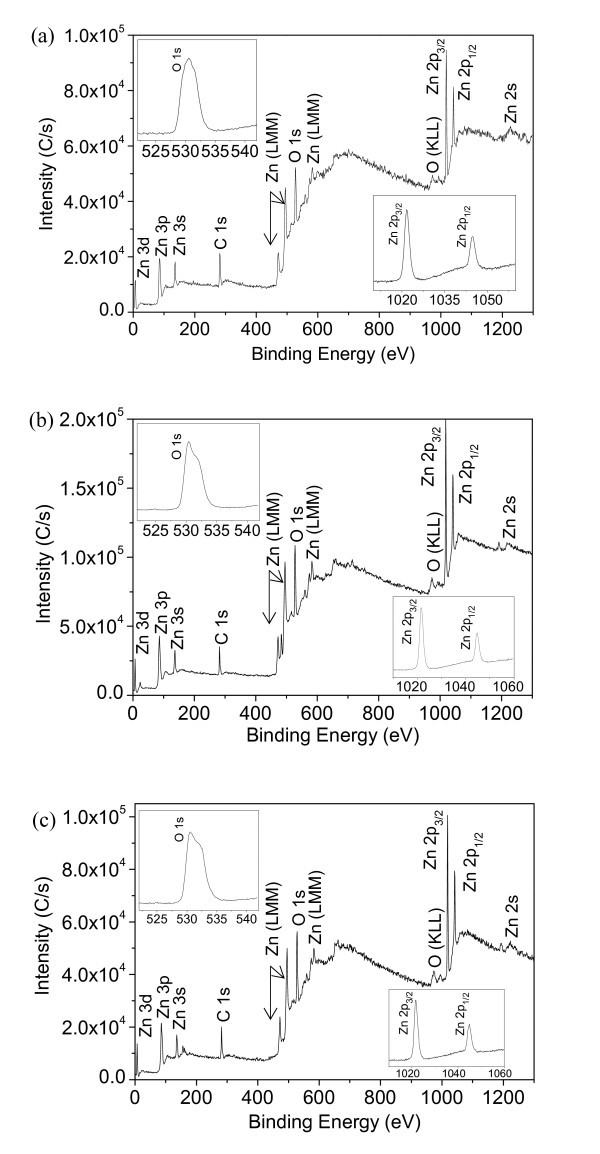
**X-ray photoelectron spectra of the as-prepared ZnO nanowire arrays**. **(a) **sample A, **(b) **sample B, and **(c) **sample C. The insets are the corresponding Zn 2p and O 1s spectra.

The surface chemical composition of all the three ZnO nanowire arrays have been characterized by means of XPS to detect any trace of impurities in the samples and to assess the vertical compositional homogeneity, as shown in Figure 2. The insets are the high resolution spectra recorded for the Zn and O regions. Binding energies were calibrated with respect to the signal for adventitious carbon with binding energy of 284.6 eV. The respective binding energies of Zn 2p_3/2 _and Zn 2p_1/2 _are all recorded with the peaks at 1021.7 and 1044.8 eV (sample A), 1021.6 and 1044.8 eV (sample B), 1021.7 and 1044.9 eV (sample C). The photoelectron spectra of O 1s in the as-prepared three samples were located at 530.6, 530.4, and 530.5 eV, respectively. The binding energies of the three samples are similar and in total agreement with the standard values of ZnO. The above XRD and XPS results revealed that our preparation method supplied pure surface compositions of all the three ZnO products, not as the same as the wet chemical approaches induced doping type in ZnO nanostructures [20,21].

**Figure 3:**
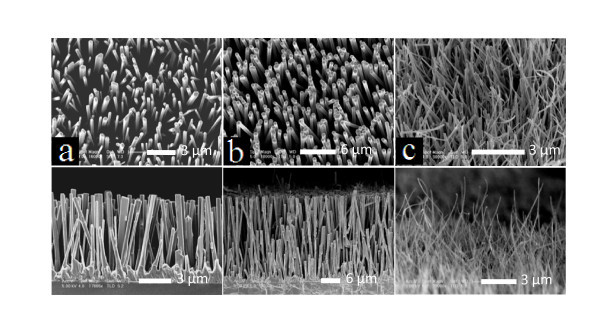
**The tilted and cross-sectional (in the corresponding below panel) FE-SEM images of (a) sample A, (b) sample B, and (c) sample C**.

**Figure 4:**
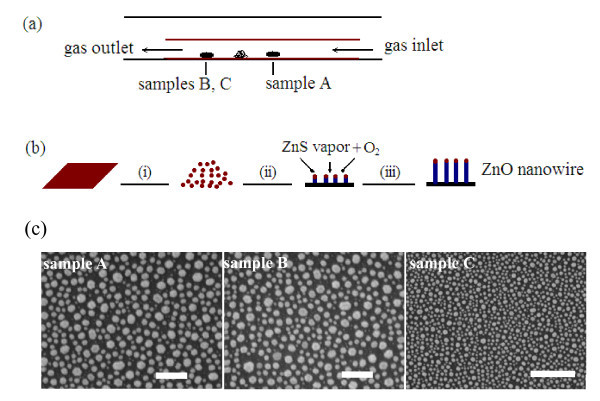
**The growth of ZnO nanowire arrays. **(a) The schematic diagram of experimental setup, (b) growth mechanism of ZnO nanowire arrays, and (c) top-view SEM images of Au catalyst on Si substrates after annealing at 750°C for 30 min in the absence of source materials. The Au film thicknesses of samples A, B and C are about 50, 50, and 15 Å, respectively. The scale bars are all 1 μm.

Figure 3 presents the quite characteristic morphologies of the three kinds of ZnO nanowire arrays, which present the tilted (the up panel) and their corresponding cross-sectional (the below panel) FE-SEM images of samples A, B, and C, respectively. The well-aligned nanowires of samples A and B are shown in large-scale, every single nanowire was self-aligned perpendicular to the silicon substrates, and there was no bending or interconnects between themselves. For the sample C, some of ZnO nanowires with small diameters with high aspect ratios are too weak to be standing up, leading to a little inclined morphology. The detailed structural parameters of samples A, B, and C are listed in Table 1. Their average diameters are about 300, 600, and 80 nm, and the corresponding lengths are 6, 25, and 25 μm, respectively. The resultant diameters and lengths in different sizes could be attributed to the thicknesses of Au thin films as well as the substrate locations (shown in Figure 4a). An underlying mechanism for morphology derivation and evolution of 1D nanostructures has been elucidated along with the advancement of preparation methods and several models that have been proposed in the previous study [22]. Here, the growth mechanism of ZnO nanowire arrays using ZnS precursor was involved based on the conventional vapor-liquid-solid (VLS) using metal catalyst as a starting material [23,24], and the schematic diagram is shown in Figure 4b. The growth stages might be briefly described as follows. Au film layers coated on Si substrates would crack to Au nanoparticles with an elevated temperature and serve as catalyst, and it reacted with ZnS vapor to form Au-Zn-S alloy liquid droplets. The alloy liquid droplets could absorb oxygen atoms, or react with oxygen atoms from air to yield ZnO molecules. Consequently, the formation of ZnO may be expressed by the corresponding chemical reaction [24]

(1) $$\text{ZnS}(\text{g})+{\text{O}}_{2}(\text{g})↔\text{ZnO}(\text{s})+{\text{SO}}_{2}(\text{g})$$

Figure 4c shows the top-view SEM images of Au-coated silicon substrates after annealing at 750°C for 30 min in the absence of source materials, but with the other experimental conditions unchanged. The Au film layer melted into separated nanoparticles with different sizes evenly distributed on the surface of Si substrates, which are about 200-300 nm in diameter for the samples A and B, but only about 40-50 nm for the sample C. It illustrates that thicker Au film layer leads to larger Au nanoparticles during the initiated growth process, in agreement with the previous study [14]. According to the VLS growth mechanism, the nanowire's diameter is defined by the Au nanoparticle's diameter, which was observed by the fact that the sample B with Au film layer about 50 Å has the nanowire with larger diameter than that of the sample C coated with Au film of 15 Å. However, diameters of all these nanowires were observed to be larger than the corresponding Au nanoparticle sizes because of the coarsening effect resulting from the formation of a supersaturated Au-Zn-S alloy liquid droplets. However, the sample A was located upstream, although it has the same Au nanoparticle size formed during the initiated growth as sample B, the captured ZnS vapor would be less than that located in the downstream, leading to insufficiency of zinc vapor so that the growth speed was decreased and the coarsening effect would not be remarkable.

### FE properties

**Figure 5:**
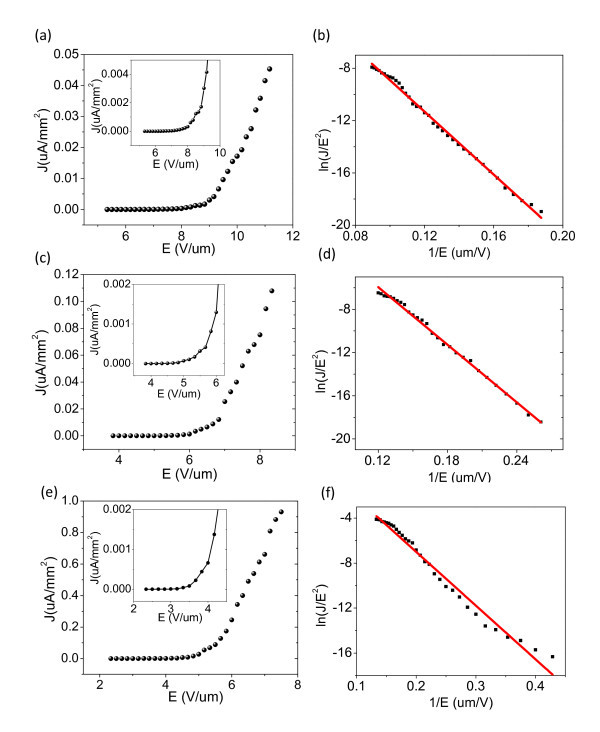
**FE properties of (a, b) sample A, (c, d) sample B, and (e, f) sample C**. The corresponding insets are the magnified parts showing the *E*_to _values clearly.

The FE properties were measured on the three kinds of ZnO nanowire arrays with different structural parameters. They were measured one after the other under exactly the same conditions. Figure 5a, c, e depicts the morphology-dependent emission current density *J *on the electric field *E *at cathode-anode distance of 300 μm for all the measurements. For the sample C, the turn-on field (*E*_to_) defined as the electric field required for reaching emission current density to 0.1 μA/cm^2 ^(0.001 μA/mm^2^) is 4.1 V/μm. It is lower than those of ZnO nanorods (5.3 V/μm) [25] and ZnO nanotubes (7.0 V/μm) [26], whereas for the samples A and B they are about 8.4 and 5.8 V/μm, respectively. The *E*_to _values can be clearly read from their corresponding insets. For further understanding of FE current-voltage characteristics, it is demonstrated by the Fowler-Nordheim (F-N) equation [27-29]

(2) $$J=(A\beta^{2}E^{2}/\Phi )\exp[-B\Phi^{3/2}{\left(\beta E\right)}^{-1}]$$

(3) $$\ln(J/E^{2})=\ln(A\beta^{2}/\Phi )-B\Phi^{3/2}/\beta E$$

where *J *and *E *are the current density, and the applied electric field, respectively. Φ is the work function of emitting materials. *A *and *B *are constants with the values of 1.56 × 10^-10 ^AeV/V^2 ^and 6.83 × 10^3 ^eV^-3/2^/μm. Figure 5b, d, f presents that the F-N lines are all have nearly linear relationship, indicating that the electron emission is indeed caused by a vacuum tunneling. β is the field-enhancement factor defined as the ratio of the local electric field at the tip of a nanowire to the macroscopic electric field, can be estimated from the slope of F-N plots. Assuming the work function of bulk ZnO to be 5.3 eV, the estimated β of the samples A, B, and C are 1209.5, 1566.7, and 1745.8, respectively. Based on the above discussions, it can be seen that the sample C has the best FE efficiency including the lowest *E*_to _and the highest β.

Many former studies have demonstrated that FE performance of ZnO nanostructured arrays can be significantly enhanced through either changing geometry configuration, achieving rational spatial distribution of the emitting centers, or increasing the aspect ratio [13,14,30]. The relationship of β and aspect ratio *l*/*r *is proposed by an empirical model [31]

(4) $$\beta =b{\left(l/r+h\right)}^{0.9}[1-\exp(-as/l)]$$

where *l*, *r*, and *s *are the length, radius, and the interspacing of ZnO nanowires, respectively; *h *is an alterable parameter which can be adjusted to fit the experimental data. It is obvious that the field-enhancement factor β can be decided by the aspect ratio and the interspacing of nanowires. The sample C has the nanowires up to 25 μm in length but only tens of nanometres in diameter; the aspect ratio as high as 312.5 could explain for its excellent FE properties. However, the aspect ratios of the samples A and B are 20 and 41.7, respectively, indicating that β is not linearly increasing with the aspect ratio, which could be attributed to the screening effect. From the experimental results, it can be observed that the *E*_to _and β values were all not proportional to their nanowire densities (revealed in Table 1), we could conclude that nanowire density was not the essence in deciding the FE efficiency of nanostructured arrays, and that it was indispensable to consider the aspect ratio including the tip morphology and the relative void ratio.

### Wetting behavior

**Figure 6:**
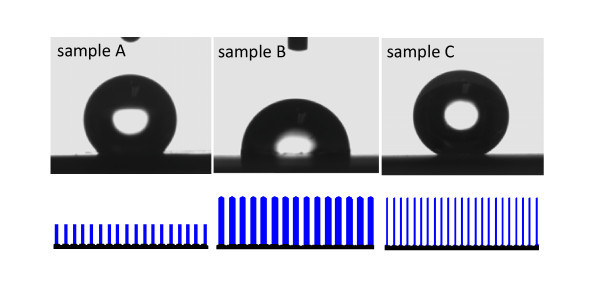
**Photographs of the measured CA values of (a) sample A, (b) sample B, and (c) sample C**. Structural schematics are shown in below panel, respectively.

Wettability was studied by examining water CA on the surfaces of three kinds of ZnO nanowire arrays. Photographs of water droplet on the three representative ZnO films with different surface morphologies are shown in Figure 6. The DI water droplets of about 5 μL were placed on the surfaces, and the CAs of the samples A and B were measured to be about 142.1° and 94.8°, respectively. However, nearly spherical droplet at the microscopic level with a measured CA value as high as 154.3° in average was obtained for the sample C, which reveals the superhydrophobic properties. The surface presents a stable character in air, with the CA showing no apparent change for up to 15 min, and the water droplet eventually evaporates on the surface of the ZnO nanowire quasi-arrays without any obvious sinking into the film. To investigate their different wetting behaviors, surface structure-induced transition may be crucial. The authors present the corresponding structural models according to the three samples (shown in Figure 5, the below panel), which clearly shows the different void ratios induced by their different diameters and interspacing of the aligned nanowires. Theoretically, a thorough understanding of the superhydrophobic phenomenon can be obtained from the Cassie and Baxter equation [32], and the CA for a composite surface is influenced greatly by the fractional areas of solid (*f*_1_) versus air pockets (*f*_2_)

(5) $$\begin{array}{cc} \cos\theta =f_{1}\cos\theta_{1}-f_{2}, & \left(f_{1}+f_{2}=1\right) \end{array}$$

Here, *θ *and *θ*_1 _are the corresponding water CAs on rough and smooth surfaces. Evidently, the CA varies with the amount of air trapped within the voids among these nanowire arrays. The nanostructured films with high void ratio would keep larger fraction of air trapped within the voids and greatly increase the air/water interface, the effectively cause the increase of water CA. For the samples A, B, and C, the void ratios are roughly calculated to be about 90.8, 83.9, and 97.9%, respectively, using the formula: *η *= (1 - *Nπr*^2^) × 100%, assuming that those nanowires for each sample have the same length and cylindrical shape. Here, *N *and *r*, respectively, represent the density (nanowires/μm^2^) and average radius of nanowires listed in Table 1. The results demonstrated a qualitative analysis that larger void ratio could play an effective approach to increase CA values for the three sample surfaces which are all ZnO nanowire arrays with same preferential orientations in the *c*-axis direction. However, decreasing the surface free energy by coating with low surface energy molecules is also greatly regarded as the other point to obtain superhydrophobic surfaces [33,34], even if the void ratio is not large enough. The sliding behavior of the sample C was also performed by fixing the sample on the platform of OCA CA system, a 5-μL water droplet was dropped on its surface and the system tilted until the water droplet rolled off. Then a SA of 7.3° in average was obtained, showing super water-repellent properties. These properties could be used for self-cleaning functions, antifog, or other fields.

## Conclusions

Three kinds of large scale ZnO nanowire arrays with different aspect ratios and void ratios were fabricated using facile thermal evaporation route using ZnS source materials. Experimental results demonstrated that ZnO nanowire arrays with larger aspect ratio and proper density have better FE properties including lower turn-on field and higher field-enhancement factors. Moreover, a larger void kept within the nanostructured films was proved to be important for preparation of super water-repellent surfaces. This study could be a good platform to elucidate the physical essence of the FE performance and wetting behavior related to the corresponding nanostructured arrays.

## Abbreviations

CA: contact angle; DI: de-ionized; FE-SEM: field emission scanning electron microscope; F-N: Fowler-Nordheim; VLS: vapor-liquid-solid.

## Competing interests

The authors declare that they have no competing interests.

## Authors' contributions

LY participated in the design of the study, carried out the total experiment, performed the statistical analysis as well as drafted the manuscript. MZ participated in the design of the study, gived the theoretical and experimental guidance, performed the statistical analysis, and gave the corrections of manuscript. LM participated in the design of experimental section and supplied the help in experiment. WL and ML mainly helped to carry out the measurement of CA and sliding angles. WS helped to amend the manuscript and the analysis of FE properties.
